# Effects of chronic sleep restriction on the neuro‐phenotypes of *Ctnnd2* knockout mice

**DOI:** 10.1002/brb3.3075

**Published:** 2023-05-24

**Authors:** Man Xu, Xiaoya Wang, Luyi Wang, Shali Wang, Jing Deng, Yan Wang, Yingbo Li, Sen Pan, Ailing Liao, Yihao Tao, Shujiang Tan

**Affiliations:** ^1^ Department of Pediatric Chongqing University Fuling Hospital Chongqing China; ^2^ Institute of Neuroscience, Department of Physiology, School of Basic Medical Science Chongqing Medical University Chongqing China; ^3^ Department of Pathology Affiliated Hospital of North Sichuan Medical College Sichuan China; ^4^ Department of Nuclear Medicine Chongqing University Fuling Hospital Chongqing China; ^5^ Department of Neurosurgery, Xinqiao Hospital Army Medical University Chongqing China; ^6^ Department of Urology Chongqing University Fuling Hospital Chongqing China; ^7^ NHC Key Laboratory of Birth Defects and Reproductive Health Chongqing Population and Family Planning Science and Technology Research Institute Chongqing China; ^8^ Department of Neurosurgery The Second Affiliated Hospital of Chongqing Medical University Chongqing China

**Keywords:** synapse, sleep restriction, dendritic spines, *Ctnnd2*, cognition, autism

## Abstract

**Introduction:**

Sleep abnormalities are highly correlated with neurodevelopmental disorders, such as intellectual disability, attention deficit hyperactivity disorder, and autism spectrum disorders (ASD). The severity of behavioral abnormalities is correlated with the presence of sleep abnormalities. Based on previous research, we investigated that *Ctnnd2* gene deletion in mice lead to ASD‐like behaviors and cognitive defects. Given the importance of sleep in individuals with ASD, this study aimed to determine the effects of chronic sleep restriction (SR) on wild‐type (WT) mice and on *Ctnnd2* deletion‐induced, neurologically related phenotypes in mice.

**Method:**

WT and *Ctnnd2* knockout (KO) mice were both subjected to manual SR (5 h per day) for 21 consecutively days separately, then we compared neurologically related phenotypes of WT mice, WT mice subjected to SR, KO mice, and KO mice subjected to SR using a three‐chamber assay, direct social interaction test, open‐field test, Morris water maze, Golgi staining, and Western blotting.

**Results:**

The effects of SR on WT and KO mice were different. After SR, social ability and cognition were impaired in both WT and KO mice. Repetitive behaviors were increased, and exploration abilities were decreased in KO mice but not in WT mice. Moreover, SR reduced the density and area of mushroom‐type dendritic spines in WT rather than KO mice. Finally, the PI3K/Akt‐mTOR pathway was found to be involved in the effects induced by SR‐impaired phenotypes in WT and KO mice.

**Conclusion:**

Overall, results of the present study may have implications for the role of disrupted sleep in patients with *CTNND2* gene‐related autism and the evolution of neurodevelopmental disorders.

## Graphical abstract

The main finding of our present study is that the deletion of *Ctnnd2* gene in mice could lead social interaction disorders, repetitive behaviors, less exploration behaviors and defects in spatial cognition. Besides, the growth of dendritic spines and synapses in prefrontal cortex(PFC) are significantly impaired in *Ctnnd2* KO mice. Next, we find that some of these phenotypes in WT and *Ctnnd2* KO mice were also influenced by chronic sleep restriction, which is similarly to that seen in human with autism. Finally, we observe that PI3K/Akt‐mTOR signal pathway was inhibited in *Ctnnd2* KO mice, and chronic sleep restriction may regulate the development of dendritic spines and synapses mediated by PI3K/Akt‐mTOR signal pathway.

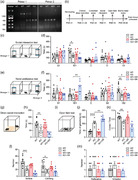

## 1 INTRODUCTION

Sleep is an intricate state in physiological in mammals, including humans, in which approximately one‐third of their lifetime is devoted to sleep (Liew & Aung, 2021). Chronic sleep disruption is very common in the modern era; during sleep disruption, an individual's working ability, cognitive function, and mood are decreased (Spiegel et al., 1999). Chronic sleep–wake disorders lead to abnormal development of the nervous system and neurobehavioral problems (Bandyopadhyay & Sigua, 2019; Sare et al., 2016), as well as various other physical and mental diseases, such as cardiovascular disease, obesity, and diabetes (Gao et al., 2019; Knutson et al., 2007; Spaeth et al., 2013). Chronic sleep disruption in adults leads to different symptoms, including daytime sleepiness, psychomotor slowing, and impairments in cognitive processing and memory (Liew & Aung, 2021). Moreover, children are more likely to be affected by a range of emotional/behavioral disturbances, including hyperactivity, emotional lability, aggressiveness, and deficits in socialization (Beebe, 2006). Furthermore, sleep‐deprived children demonstrate difficult behaviors, which can be stressful and negatively impact on the quality of life for the entire family.

Some diseases, such as autism spectrum disorder (ASD), are frequently associated with comorbidities including sleep problems. ASD is one of the most common neurodevelopmental disorders and is characterized by core deficits in social communication, interaction defects, and repetitive behaviors (de la Torre‐Ubieta et al., 2016). Studies have reported high prevalence rates, reaching 86% of sleep disorders in children with ASD (Gisbert Gustemps et al., 2021), and numerous studies support the possibility that insufficient sleep exacerbates the behavioral symptoms of ASD (Missig et al., 2020). For example, among children with autism, sleep–wake disturbances predict greater severity of core ASD behaviors, such as deficits in social skills (Missig et al., 2020), exacerbate some externalizing behavioral problems, such as aggression and impulsivity (Sikora et al., 2012), and even deteriorate the cognition ability. Moreover, although sleep problems are believed to worsen behavior, the inverse may also be true, with behavioral problems worsening sleep problems (Gisbert Gustemps et al., 2021). Nevertheless, the specific relationship between sleep and ASD‐like behaviors remains unclear.

Chronic sleep–wake disorders may result in disrupted synaptic plasticity, such as impaired myelination, synapse formation/function dysfunction, and abnormalities in synapse‐related protein synthesis (Areal et al., 2017; Cirelli & Tononi, 2020; Picchioni et al., 2014). In addition, altered structures of dendritic spines and synaptic function are major hallmarks of patients with ASD reported in previous studies (Bagni & Zukin, 2019). For example, increased spine density has been observed in brain tissues from humans with ASD, whereas an increase in spine density on apical dendrites of pyramidal neurons in some but not all cortical brain areas has been detected (Hutsler & Zhang, 2010). Furthermore, reduced developmental spine pruning in layer V pyramidal neurons in the postmortem ASD temporal lobe has been reported to be correlated with hyperactivated mTOR and impaired autophagy (Tang et al., 2014).

Currently, *CTNND2* (delta‐catenin) has attracted much attention from researchers regarding its relationship with the occurrence of ASD. A study published in Nature first investigated the association between δ‐catenin and potentially crucial roles in severe ASD (Turner et al., 2015). A case report described a 5‐year‐old male child with developmental delay, behavioral problems, and dysmorphic features, who was found by microarray to harbor a 93‐kb duplication of uncertain significance that fully encompasses the third exon of *CTNND2*. This was used to determine whether the duplication was tandem and predicted to lead to *CTNND2* haploinsufficiency (Miller et al., 2020). Moreover, *CTNND2* is usually deleted in individuals with cri‐du‐chat syndrome, a disorder classically defined by intellectual disability (Wu et al., 2005). Intragenic *CTNND2* deletion was detected using molecular karyotyping in two patients with isolated intellectual disability (Belcaro et al., 2015). Our previous study was the first to establish *Ctnnd2* knockout (KO) mice using CRISPR/Cas9 technology and demonstrated that *Ctnnd2* KO mice exhibited core symptoms of ASD, impaired learning and memory function, and synaptic dendritic spines growth retardation (Wang et al., 2021). Given the important role of sleep in patients with ASD, it is worthwhile to explore the relationship between chronic sleep disruption and ASD in *Ctnnd2* KO mice.

In our study, we repeated some behavioral tests to verify the above phenotypes in *Ctnnd2* KO mice again. We hypothesized that chronic sleep restriction (SR) would further exacerbate the autism‐like behaviors, cognition, and growth of dendritic spines and synapses in *Ctnnd2* KO mice. At the same time, we also evaluated the effects of SR on wild‐type (WT) mice and discussed the different influence of SR in WT and KO mice. Moreover, recently studies have focused on the relationship between the PI3K/Akt‐mTOR signaling pathway and sleep, and have reported that SR decreased the phosphorylation of PI3K and Akt (Xue et al., 2019; Yan et al., 2019). Accordingly, we examined the underlying molecular mechanisms underlying SR in WT and *Ctnnd2* KO mice.

## 2 MATERIALS AND METHODS

### 2.1 Animal and care

*Ctnnd2* KO mice were originally obtained by CRISPR/Cas9‐mediated genome engineering technology in collaboration with the Nanjing Institute of Biomedicine at Nanjing University and maintained in the Animal Core Facility of Chongqing Medical University (Chongqing, China). By crossing *Ctnnd2* KO mice with C57BL/6J mice, we got heterozygous (HET) *Ctnnd2* KO mice, and by crossing HET *Ctnnd2* KO mice, we got WT, HET, and homozygote KO mice. Only male WT and homozygote KO were used in the experiments.

After weaning at 3 weeks, mice were group‐housed with 4–6 mice per cage under a controlled environment (22 ± 2°C, 45% ± 10% humidity, 12 h light/dark cycle, lights from 6 a.m. to 6 p.m.) with free access to water and food. WT mice used for three‐chamber sociability and direct social interaction test were male, conspecific, and aged 7–8 weeks. Appropriate measures were performed to reduce the pain and discomfort of experimental animals. The animal protocols were evaluated and approved by the Ethics Committee of Chongqing Medical University (protocol no. 2015‐051).

### 2.2 Genotyping

**FIGURE 1:**
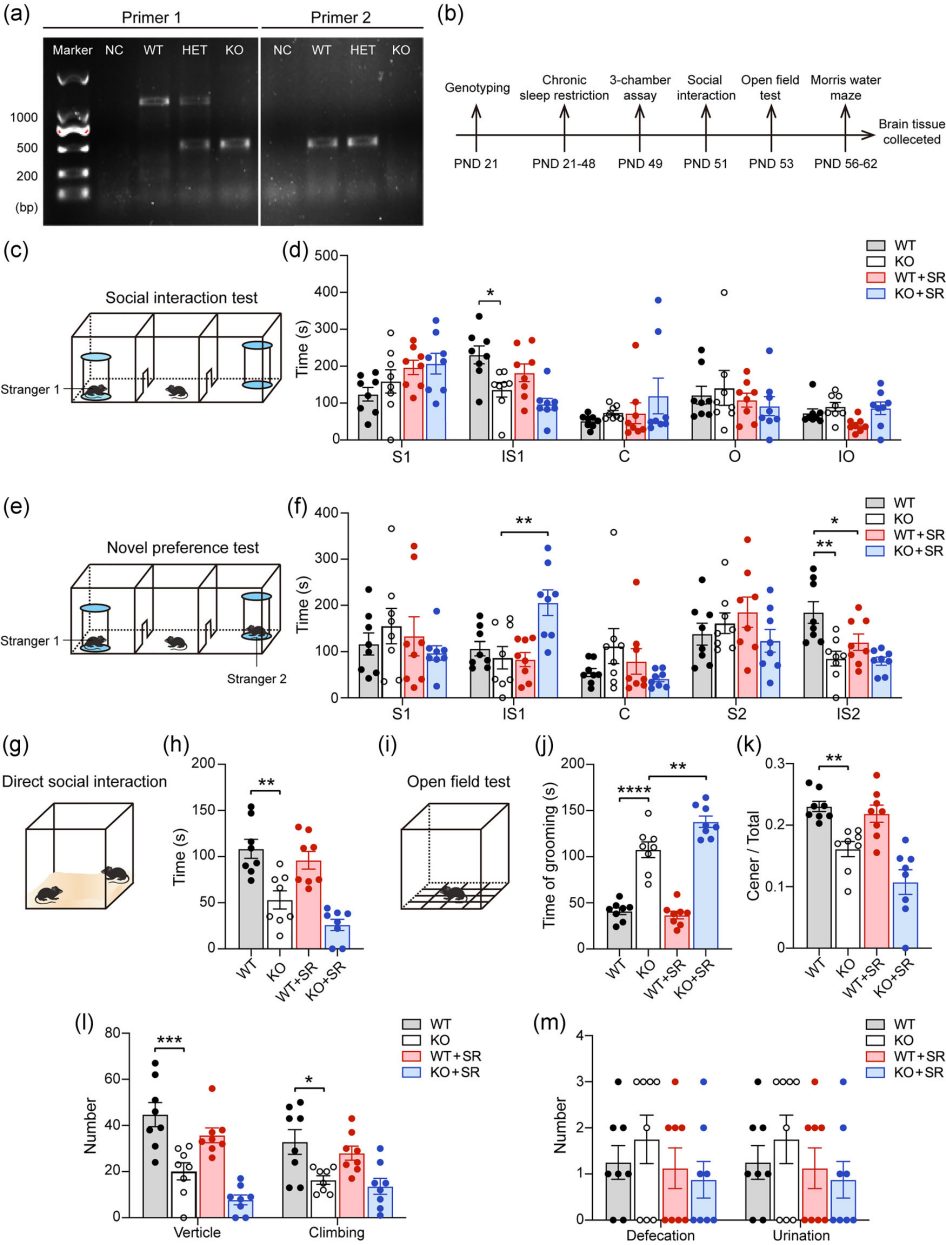
**The results of autism‐like behaviors, anxiety, and exploration behaviors of four groups mice**: **(a)** genotyping results of wild type (WT), heterozygote (HET), and knockout (KO) mice; **(b)** timelines of experiments; **(c–f)** three‐chamber assay of four groups mice. Schematic presentation **(c)** and statistical analysis **(d)** of social interaction test. Schematic presentation **(e)** and statistical analysis **(f)** of novelty preference test. **(g and h)** Schematic presentation **(g)** and statistical analysis of time in direct social interaction **(h)**. **(i–m)** Open‐field test of four groups mice. Schematic presentation **(i)** and statistical analysis of time of grooming **(j)**, ratio of center/total grids **(k)**, frequency of vertical and climbing **(l),** and number of excretions **(m)**. PND, postnatal; S1, time of test mouse staying in stranger 1 mouse chamber without interaction with stranger 1; IS1, time of test mouse interaction with Stranger 1; C, time of test mouse staying center chamber; O, time of test mouse staying in object chamber, IO, time of test mouse interaction with object; S2, time of test mouse staying in stranger 2 mouse chamber withour interaction with stranger 2; IS2, time of test mouse interaction with Stranger 2; SR, sleep restriction. All groups: *n* = 8. Data are the means ± SEM. *p < .05, **p < .01, ***p < .001, and ****p < .0001.

To determine the genotypes (WT, HET, and KO) of mice, a single tail snip was collected from mice at the time of weaning (postnatal day, PND 21), and DNA fragments were amplified using polymerase chain reaction with two sets of primers were used: one is 5′‐TTCTGTATTTCACAGTACCAAC‐3′, 5′‐AACTCATCATAAGAAACACCTG‐3′, another is 5′‐TGTTTGACTTCATTGTTACAG‐3′ and 5′‐CAACTGTCACCCTACTTTAGT‐3′ (Figure 1a).

### 2.3 Three‐chamber sociability assay

A three‐chamber box was constructed from transparent plastic boards and measuring 120 cm × 20 cm × 22 cm, which was divided by baffles into three equal chambers, as previously described (Hou et al., 2018; Wang et al., 2021). The two side chambers contained small wire cages to later house social mice. The movements were tracked and recorded using a video recorder.

Briefly, mice were habituated for 10 min and allowed to freely explore all three chambers before the test. Then, a male conspecific juvenile mouse at the same age (Stranger 1) was introduced into the left wire mesh cup. In the first 10 min of the social interaction test, the test mouse was placed in the center chamber without baffles. The wire mesh cup in the right chamber was considered the object. The time of test mouse spent in the left chamber without interaction with Stranger 1 (tested mouse actively sniffing and touching the mesh cup) (S1), spent in the interaction with Stranger 1 (IS1), spent in the center chamber (C), spent in the right chamber without interaction with object (O), and spent in the interaction with the object (IO) were manually recorded separately.

A novelty preference test was conducted during the second 10 min of the session. Another male conspecific mouse at the same age (Stranger 2) was placed into the wire mesh cup in the right chamber. The time of test mouse spent in the left chamber without interaction with Stranger 1 (tested mouse actively sniffing and touching the mesh cup) (S1), spent in the interaction with Stranger 1 (IS1), spent in the center chamber (C), spent in the right chamber without interaction with Stanger 2 (S2), and spent in the interaction with the Stranger 2 (IS2) were manually recorded separately. These data were used to determine the preference of the tested mouse for interaction with the familiar or novel animal. The apparatus was cleaned with 75% ethanol and water between tests. After the three‐chamber sociability assay, mice were allowed to relax in their home cages for 48 h.

### 2.4 Direct social interaction test

Direct social interaction test was conducted in a transparent cage (40 × 40 × 30 cm^3^) with clean padding (thickness: 2–3 cm). Mice were allowed to explore the cage freely for 10 min (habituation) before an unfamiliar conspecific male mouse was introduced into the cage. Measured the time of social interaction behaviors included body sniffing, anogenital sniffing, direct contact, and close following (<1 cm) initiated by the test mouse for 10 min. Padding in the cage was replaced before the next test.

### 2.5 Open‐field test

The open‐field test was conducted in a transparent cage (40 × 40 × 30 cm^3^) contained 16 blocks of equal size, and the 4 blocks in the center were the center grid. Mice were habituated 10 min in the cage before test and then placed in the center block again. Mice were allowed to explore free for 10 min and monitored with a video camera. The time of grooming, the number of blocks that pups passed through (cross grid), the frequency of straight upward movements (climbing and vertical), and the number of excretions (defecation and urination) were recorded separately. The open field was thoroughly cleaned with 75% alcohol and water before the next test was done for another mouse.

### 2.6 Morris water maze (MWM)

The learning and memory function of mice was evaluated by the MWM test as described previously (Cao et al., 2019; Wang et al., 2021). The MWM includes a submerged platform (10 cm × 10 cm) located within a circular black pool (diameter 120 cm) and is filled with opaque water mixed with titanium dioxide at 22 ± 1°C.

In the place navigation test, the mice were tested for their ability to find the platform for five consecutive days. The time taken to find the platform was recorded as latency. The platform was constantly but the mice were placed in a novel starting position of the maze, facing the tank wall. If the mouse did not locate the platform within 60 s, it was gently guided to it and allowed to stay for 15 s.

A spatial probe trial was conducted on the sixth day. By removing the platform and placing the mice in a position opposite to the target quadrant, the animal was allowed to swim freely for 60 s. The swimming time in the target quadrant, the number of times crossing the target, and the total distances mice swimming were recorded. The moving track of mice was recorded by a tracking system connected to an image analyzer (HVS Image, Hampton, UK) in a double‐blind manner.

### 2.7 Sleep restriction (SR)

SR was achieved using a continuously monitored enriched environment in which novel climbing toys were periodically exchanged or gentle patting cages whenever a mouse became behaviorally quiescent (Lemons et al., 2018; Sare et al., 2016, 2019). In addition, the experiment was performed in the home cages. The KO mice were sleep deprived once daily from ZT5 to ZT10 for 28 consecutive days before behavioral tests. All mice were habituated to the presence of the experimenter and for 3 days prior to the SR experiment.

### 2.8 Golgi impregnation

Golgi impregnation of prefrontal cortex (PFC) in mice was performed with the FD Rapid GolgiStainTM Kit (FD NeuroTechnologies) following the manufacturer's protocol. After behavioral tests completed, mice were anesthetized by pentobarbital, and the brains of the mice were rapidly removed and were flushed with saline. Brains were then immersed in a Golgi–Cox mixture solution (1:1, v/v) of A and B at room temperature for 2 weeks and then immersed in solution C stored at 4°C for another 72 h. After that, brains were dissected into 100‐μm sections using a cryostat microtome (Leica CM1860) and were mounted on gelatin‐coated slides and dried naturally for staining. The secondary or tertiary dendrites of PFC neurons were visualized using an Eclipse E100 microscope (Nikon) and measured using ScopeImage software (version 9.0).

### 2.9 Western blotting

Mice were anesthetized with pentobarbital and intracardially perfused with 0.9% saline, then the brains were removed. The PFC tissue was lysed by RIPA lysis buffer (P0013B, Beyotime, Shanghai, China) and centrifuged at 12,000 r for 15 min (4°C). A protein assay kit (P0010, Beyotime, Shanghai, China) was used to determine the protein concentrations. Then the proteins was separated with SDS–PAGE (P0012A, Beyotime, Shanghai, China) and transformed into PVDF membranes. After blocking with 5% nonfat milk powder for 2 h, the membranes were incubated with primary antibodies at 4°C overnight: anti‐ELKS (1:2000, ab180507, Abcam, USA), anti‐PSD95 (1:8000, 3450, Cell Signaling Technology, USA), anti‐p‐synapsin (1:50,000, ab76260, Abcam, USA), anti‐synapsin (1:100,000, 5297, Cell Signaling Technology, USA), anti‐p‐mTOR (1:2000, 5536, Cell Signaling Technology, USA), anti‐mTOR (1:1000, 2983, Cell Signaling Technology, USA), anti‐p‐PI3K (1:1000, AP0854, ABclonal, Wuhan, China), anti‐PI3K (1:2000, 4257, Cell Signaling Technology, USA), anti‐p‐Akt (Ser473) (1:3000, AF0016, Affinity Biosciences, USA), anti‐p‐Akt (Thr308) (1:3000, AF3262, Affinity Biosciences, USA), anti‐AKT (1:2000, 9272, Cell Signaling Technology, USA), and anti‐β‐actin (1:6000, 20536‐1‐AP, Proteintech, Wuhan, China). After extensive washing with TBST, the blots were incubated with the appropriate peroxidase‐labeled secondary antibody (1:5000, Beyotime, Shanghai, China) for 1 h in the TBST‐dry milk buffer at room temperature. Bands were scanned and densitometrically analyzed by automated ImageJ software (NIH Image, Version 1.61), the indicated total proteins were expressed relative to β‐actin signals.

### 2.10 Statistical analysis

All data were presented as mean ± SEM and statistical analyses were carried out using Graphpad Prism 9.0 and SPSS 27.0 software. Differences among four groups (indexes from three‐chamber assay, direct social interaction, open‐field test, day 6 of MWM, analyses of dendritic spines and expressions of proteins) were analyzed by means of a Two‐way ANOVA test with genotype (WT or KO) and condition (normal sleep or SR) followed by Tukey's multiple comparisons test. Specially, data from MWM (escape latency and swim speed) were analyzed by means of a Three‐way ANOVA test with genotype (WT or KO) as a between‐subject variable, condition (normal sleep or SR), and day (day 1–5) as within‐subject variables followed by Tukey's multiple comparisons test. Effects with *p* < .05 were considered statistically significant and were indicated with an “*” (Tables 1, 2, 3, 4, 5). Tables reporting *F*‐values and corresponding *p*‐values for interactions and main effects are presented for all the ANOVA data (Tables 1, 2, 3, 4, 5). All tests and measurements were performed blind to the genotype or treatment.

**TABLE 1:** Two‐way ANOVA test followed by repeated comparison of autism‐like behaviors.

Test	Interaction	Main effect	*F*_(df, error)_ value	*p*‐Value	Partial *η* ^2^
Three‐chamber assay (in the first 10 min)					
**S1**	Genotype × condition		*F*_(1, 28)_ = 0.2438	.6253	.009
		Genotype	*F*_(1, 28)_ = 0.8186	.3733	.028
		Condition	*F*_(1, 28)_ = 5.874	.0221*	.173
**IS1**	Genotype × condition		*F*_(1, 28)_ = 0.05168	.8218	.002
		Genotype	*F*_(1, 28)_ = 17.95	.0002*	.391
		Condition	*F*_(1, 28)_ = 4.284	.0478*	.133
**C**	Genotype × condition		*F*_(1, 28)_ = 0.1867	.6690	.007
		Genotype	*F*_(1, 28)_ = 1.523	.2274	.052
		Condition	*F*_(1, 28)_ = 1.395	.2476	.047
**O**	Genotype × condition		*F*_(1, 28)_ = 0.3401	.5645	.012
		Genotype	*F*_(1, 28)_ = 1.022	.9601	.0001
		Condition	*F*_(1, 28)_ = 0.002545	.3207	.035
**IO**	Genotype × condition		*F*_(1, 28)_ = 1.289	.2659	.044
		Genotype	*F*_(1, 28)_ = 6.473	.0168*	.188
		Condition	*F*_(1, 28)_ = 2.131	.1555	.071
**Total entries**	Genotype × condition		*F*_(1, 28)_ = 1.055	.3132	.036
		Genotype	*F*_(1, 28)_ = 0.07090	.7920	.003
		Condition	*F*_(1, 28)_ = 0.000	>.9999	.036
Three‐chamber assay (in the second 10 min)					
**S1**	Genotype × condition		*F*_(1, 28)_ = 1.380	.2501	.047
		Genotype	*F*_(1, 28)_ = 0.001880	.9657	.0001
		Condition	*F*_(1, 28)_ = 0.4333	.5158	.015
**IS1**	Genotype × condition		*F*_(1, 28)_ = 10.98	.0026*	.282
		Genotype	*F*_(1, 28)_ = 5.788	.0230*	.171
		Condition	*F*_(1, 28)_ = 4.901	.0352*	.149
**C**	Genotype × condition		*F*_(1, 28)_ = 3.907	.0580	.122
		Genotype	*F*_(1, 28)_ = 0.1613	.6910	.006
		Condition	*F*_(1, 28)_ = 0.9512	.3378	.033
**S2**	Genotype × condition		*F*_(1, 28)_ = 2.673	.1133	.087
		Genotype	*F*_(1, 28)_ = 0.5471	.4657	.019
		Condition	*F*_(1, 28)_ = 0.03223	.8588	.001
**IS2**	Genotype × condition		*F*_(1, 28)_ = 3.283	.0807	.105
		Genotype	*F*_(1, 28)_ = 16.70	.0003*	.374
		Condition	*F*_(1, 28)_ = 4.571	.0414*	.140
**Total entries**	Genotype × condition		*F*_(1, 28)_ = 0.2537	.6184	.009
		Genotype	*F*_(1, 28)_ = 0.06732	.7972	.002
		Condition	*F*_(1, 28)_ = 0.06732	.7972	.002
Direct social interaction					
**Social time**	Genotype × condition		*F*_(1, 28)_ = 0.6543	.4254	.023
		Genotype	*F*_(1, 28)_ = 47.18	<.0001*	.628
		Condition	*F*_(1, 28)_ = 4.692	.0390*	.144
Open field					
**Self‐grooming**	Genotype × condition		*F*_(1, 28)_ = 8.345	.0074*	.230
		Genotype	*F*_(1, 28)_ = 199.0	<.0001*	.877
		Condition	*F*_(1, 28)_ = 4.820	.0366*	.147
**Center / total**	Genotype × condition		*F*_(1, 28)_ = 2.164	.1524	.072
		Genotype	*F*_(1, 28)_ = 39.87	<.0001*	.587
		Condition	*F*_(1, 28)_ = 5.246	.0297*	.158
**Vertical**	Genotype × condition		*F*_(1, 28)_ = 0.2038	.6551	.007
		Genotype	*F*_(1, 28)_ = 49.56	<.0001*	.639
		Condition	*F*_(1, 28)_ = 8.176	.0079*	.226
**Climbing**	Genotype × condition		*F*_(1, 28)_ = 0.08456	.7734	.003
		Genotype	*F*_(1, 28)_ = 17.85	.0002*	.389
		Condition	*F*_(1, 28)_ = 1.089	.3057	.037
**Defecation**	Genotype × condition		*F*_(1, 28)_ = 0.7368	.3980	.026
		Genotype	*F*_(1, 28)_ = 0.08187	.7769	.003
		Condition	*F*_(1, 28)_ = 1.31	.2621	.045
**Urination**	Genotype × condition		*F*_(1, 28)_ = 0.08642	.7709	.003
		Genotype	*F*_(1, 28)_ = 2.160	.1527	.072
		Condition	*F*_(1, 28)_ = 0.7778	.3853	.027

**TABLE 2:** Three‐way ANOVA test followed by repeated comparison of Morris water maze (MWM).

Test	Effect	Main effect	*F*_(df, error)_ value	*p*‐Value	Partial *η* ^2^
MWM (day 1–5)					
**Escape latency**	Genotype × condition × day		*F*_(4, 140)_ = 0.02108	.9991	.002
	Genotype × condition		*F*_(1, 140)_ = 0.8545	.3569	.052
	Genotype × day		*F*_(4, 140)_ = 10.36	<.0001*	.369
	Condition × day		*F*_(4, 140)_ = 2.690	.0336*	.220
		Genotype	*F*_(1, 140)_ = 134.8	<.0001*	.897
		Condition	*F*_(1, 140)_ = 38.48	<.0001*	.714
		Day	*F*_(4, 140)_ = 290.4	<.0001*	.942
**Swim speed**	Genotype × condition × day		*F*_(4, 140)_ = 0.0.06196	.9928	.005
	Genotype × condition		*F*_(1, 140)_ = 0.1193	.7303	.014
	Genotype × day		*F*_(4, 140)_ = 0.1696	.9536	.011
	Condition × day		*F*_(4, 140)_ = 0.2485	.9102	.019
		Genotype	*F*_(1, 140)_ = 0.09382	.7598	.005
		Condition	*F*_(1, 140)_ = 0.08775	.7675	.010
		Day	*F*_(4, 140)_ = 0.1847	.9460	.012

**TABLE 3:** Two‐way ANOVA test followed by repeated comparison of Morris water maze (MWM).

Test	Effect	Main effect	*F*_(df, error)_ value	*p*‐Value	Partial *η* ^2^
MWM (day 6)					
**Number of passing**					
**through platform**	Genotype × condition		*F*_(1, 28)_ = 0.1189	.7328	.004
		Genotype	*F*_(1, 28)_ = 52.43	<.0001*	.652
		Condition	*F*_(1, 28)_ = 18.58	.0002*	.399
**Time in target zone**	Genotype × condition		*F*_(1, 28)_ = 0.01553	.9017	.001
		Genotype	*F*_(1, 28)_ = 66.56	<.0001*	.704
		Condition	*F*_(1, 28)_ = 27.15	<.0001*	.492
**Total distance**	Genotype × condition		*F*_(1, 28)_ = 0.1801	.6745	.006
		Genotype	*F*_(1, 28)_ = 0.1099	.7428	.004
		Condition	*F*_(1, 28)_ = 0.2473	.6229	.009

**TABLE 4:** Two‐way ANOVA test followed by repeated comparison of dendritic spines.

Dendritic spines	Effect		*F*_(df, error)_ value	*p*‐Value	Partial *η* ^2^
Dendritic spines density					
**Total**	Genotype × condition		*F*_(1, 104)_ = 0.6547	.4203	.006
		Genotype	*F*_(1, 104)_ = 243.9	<.0001*	.701
		Condition	*F*_(1, 104)_ = 4.431	.0377*	.041
**Mushroom**	Genotype × condition		*F*_(1, 104)_ = 2.415	.1232	.023
		Genotype	*F*_(1, 104)_ = 276.6	<.0001*	.727
		Condition	*F*_(1, 104)_ = 13.68	.0003*	.116
**Stubby**	Genotype × condition		*F*_(1, 104)_ = 1.951	.1655	.018
		Genotype	*F*_(1, 104)_ = 62.47	<.0001*	.375
		Condition	*F*_(1, 104)_ = 0.9203	.3396	.009
**Filopodia**	Genotype × condition		*F*_(1, 104)_ = 1.599	.2089	.015
		Genotype	*F*_(1, 104)_ = 5.628	.0195*	.051
		Condition	*F*_(1, 104)_ = 0.1946	.6600	.002
Dendritic spines area					
**Mushroom**	Genotype × condition		*F*_(1, 104)_ = 1.637	.2036	.015
		Genotype	*F*_(1, 104)_ = 204.5	<.0001*	.663
		Condition	*F*_(1, 104)_ = 10.38	.0017*	.091
**Stubby**	Genotype × condition		*F*_(1, 104)_ = 0.09483	.7587	.001
		Genotype	*F*_(1, 104)_ = 65.04	<.0001*	.385
		Condition	*F*_(1, 104)_ = 3.114	.0806	.029
**Filopodia**	Genotype × condition		*F*_(1, 104)_ = 4.963	.0281*	.046
		Genotype	*F*_(1, 104)_ = 3.668	.0582	.034
		Condition	*F*_(1, 104)_ = 0.9180	.0582	.009

**TABLE 5:** Two‐way ANOVA test followed by repeated comparison of western blot.

Protein	Effect		*F*_(df, error)_ value	*p*‐Value	Partial *η* ^2^
**ELKS/β‐actin**	Genotype × condition		*F*_(1, 32)_ = 0.3890	.5372	.012
		Genotype	*F*_(1, 32)_ = 34.03	<.0001*	.515
		Condition	*F*_(1, 32)_ = 20.10	<.0001*	.386
**PSD95/β‐actin**	Genotype × condition		*F*_(1, 32)_ = 0.4446	.5097	.014
		Genotype	*F*_(1, 32)_ = 39.25	<.0001*	.551
		Condition	*F*_(1, 32)_ = 20.38	<.0001*	.389
**p‐synapsin/synapsin**	Genotype × condition		*F*_(1, 32)_ = 0.3672	.5488	.011
		Genotype	*F*_(1, 32)_ = 138.7	<.0001*	.813
		Condition	*F*_(1, 32)_ = 20.13	<.0001*	.386
**p‐mTOR/mTOR**	Genotype × condition		*F*_(1, 32)_ = 0.004908	.9446	.0001
		Genotype	*F*_(1, 32)_ = 49.25	<.0001*	.606
		Condition	*F*_(1, 32)_ = 15.70	.0004*	.329
**p‐PI3K/PI3K**	Genotype × condition		*F*_(1, 32)_ = 7.082e − 005	.9933	.0001
		Genotype	*F*_(1, 32)_ = 110.6	<.0001*	.776
		Condition	*F*_(1, 32)_ = 21.15	<.0001*	.398
**p‐Akt (473)/Akt**	Genotype × condition		*F*_(1, 32)_ = 0.3781	.5430	.012
		Genotype	*F*_(1, 32)_ = 47.88	<.0001*	.599
		Condition	*F*_(1, 32)_ = 24.36	<.0001*	.432
**p‐Akt (308)/Akt**	Genotype × condition		*F*_(1, 32)_ = 0.3528	.5567	.011
		Genotype	*F*_(1, 32)_ = 58.36	<.0001*	.646
		Condition	*F*_(1, 32)_ = 23.36	<.0001*	.422

## 3 RESULTS

### 3.1 Effects of SR on autism‐like behaviors in WT and *Ctnnd2* KO mice

To examine the effects of SR on the response to autism‐like behaviors, a series of behavioral tests were performed after WT and KO mice subjected to chronic SR. To examine sociability, in the three‐chamber assay, only one stranger mouse (Stranger 1) was placed in the left chamber during the first 10 min. Compared with WT mice, KO mice spent less time interacting with Stranger 1 (*p* = .0188, Figure 1c,d). After SR, both WT and KO mice exhibited less time interaction with Stranger 1; however, there was no significantly difference between them (Figure 1c,d). Next, to examine the animals’ interest in social novelty, a second mouse (Stranger 2) was placed in the right chamber during the second 10 min. KO mice spent less time interacting with Stranger 2 (*p* = .0014, Figure 1e,f). SR reduced the time interaction time with Stranger 2 in both WT and KO mice; however, there was a significantly difference only between the WT and WT+SR groups (*p* = .0435, Figure 1e,f). KO mice subjected to SR spent more time with Stranger 1 in the second 10 min (*p* = .0029, Figure 1e,f). In the direct social interaction test, only KO mice exhibited reduced interaction time with the stranger mice (*p* = .0011, Figure 1g,h). In the open‐field test, KO mice exhibited stereotypic and repetitive behaviors with more self‐grooming time (*p* < .0001, Figure 1j) than WT mice. At the same time, KO mice exhibited impaired exploration ability due to a lower ratio of crossing center/total grids (*p* = .0098, Figure 1k) and the decreased number of vertical (*p* = .0004) and climbing (*p* = .0172) movements (Figure 1l). Next, it was found that SR only increased the self‐grooming time (*p* = .0064, Figure 1j) of KO mice during the open‐field test. Finally, to further observe the anxiety in the mice, a number of defecation and urination episodes were counted, with no differences among them (Figure 1m). Collectively, these data suggested that the effects of SR on autism‐like behaviors in WT and *Ctnnd2* KO mice were not the same. SR influenced the social novel preference of WT and *Ctnnd2* KO mice and only deteriorated the stereotypic and repetitive behaviors of *Ctnnd2* KO mice.

### 3.2 SR deteriorated the learning and memory function in WT and *Ctnnd2* KO mice

**FIGURE 2:**
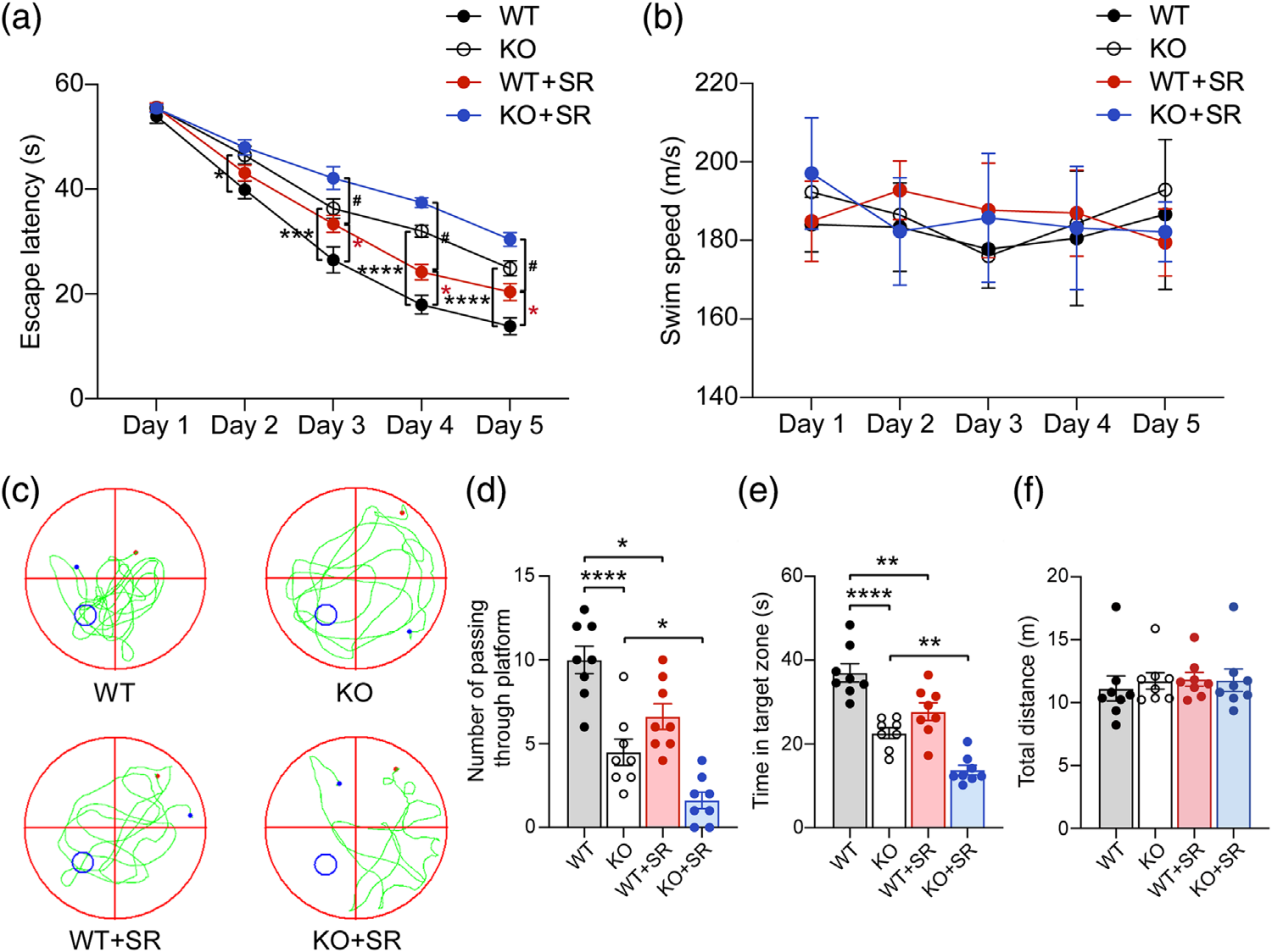
**The spatial learning and memory ability of four groups mice**: **(a)** escape latency time to find the submerged platform from day 1 to 5 of learning phase; **(b)** average swim speed from day 1 to 5; **(c)** representative swimming traces were shown for probe test of four groups mice; **(d–f)** the number of platform crossings **(d)**, the time in the target zone **(e),** and the total distances **(f)** during the probe trial on day six were analyzed to evaluate the mice's spatial memory. WT, wild type; KO, knockout; SR, sleep restriction. All groups: *n* = 8. *p < .05, **p < .01, ***p < .001, ****p < .0001, and ^#^ *p* < .05.

The MWM was used to evaluate the spatial learning and memory ability of the mice. In the learning phase, mice were permitted to train for 5 days to explore and find the location of the hidden platform. The time taken by the mice to find the hidden platform was defined as escape latency and swim speed was recorded to evaluate locomotor ability. KO mice spent more time finding the hidden platform, and multiple comparisons revealed that the time spent by KO mice exploring the hidden platform significantly increased on days 2–5 (day 2: *p* = .0284, day 3: *p* = .0004, day 4: *p* < .0001, and day 5: *p* < .0001) relative to WT mice (Figure 2a). At the same time, compared with WT mice, WT mice subjected to SR spent more time to find the hidden platform, and multiple comparisons revealed that the time of the WT mice subjected to SR spent finding the hidden platform significantly increased on days 3–5 (day 3: *p* = .0208, day 4: *p* = .0433, and day 5: *p* = .0314, Figure 2a). Moreover, compared with KO mice, SR increased the time of the mice spent finding the hidden platform, and the time spent finding the hidden platform significantly increased on days 3–5 (day 3: *p* = .0274, day 4: *p* = .0431, and day 5: *p* = .0408) for KO mice subjected to SR analyzed by multiple comparisons (Figure 2a). There was no significantly difference in swim speed of any of the groups of mice (Figure 2b).

During the memory test, the number of platform crossings was counted and the time spent in the target quadrant by observing track events of the mice in the pool without a platform. Compared to WT mice, KO mice crossed the platform less often (*p* < .0001, Figure 2c,d) and spent less time in the target quadrant (*p* < .0001, Figure 2c,e). WT mice subjected to SR conditions also crossed the platform less often (*p* = .0135, Figure 2c,d) and spent less time in the target quadrant (*p* = .0041, Figure 2c,d). Furthermore, relative to KO mice, the number of platform crossings (*p* = .0425, Figure 2c,d) and the time spent in the target quadrant (*p* = .0064, Figure 2c,d) decreased in KO mice that experienced SR. Finally, the total distance traveled by the mice in the pool was recorded, with no significantly differences found (Figure 2e). In summary, these data suggested that SR deteriorated the learning and memory function in WT and *Ctnnd2* KO mice.

### 3.3 SR affects dendritic spine morphology and expression of synapse‐related protein in the PFC of WT and *Ctnnd2* KO mice

**FIGURE 3:**
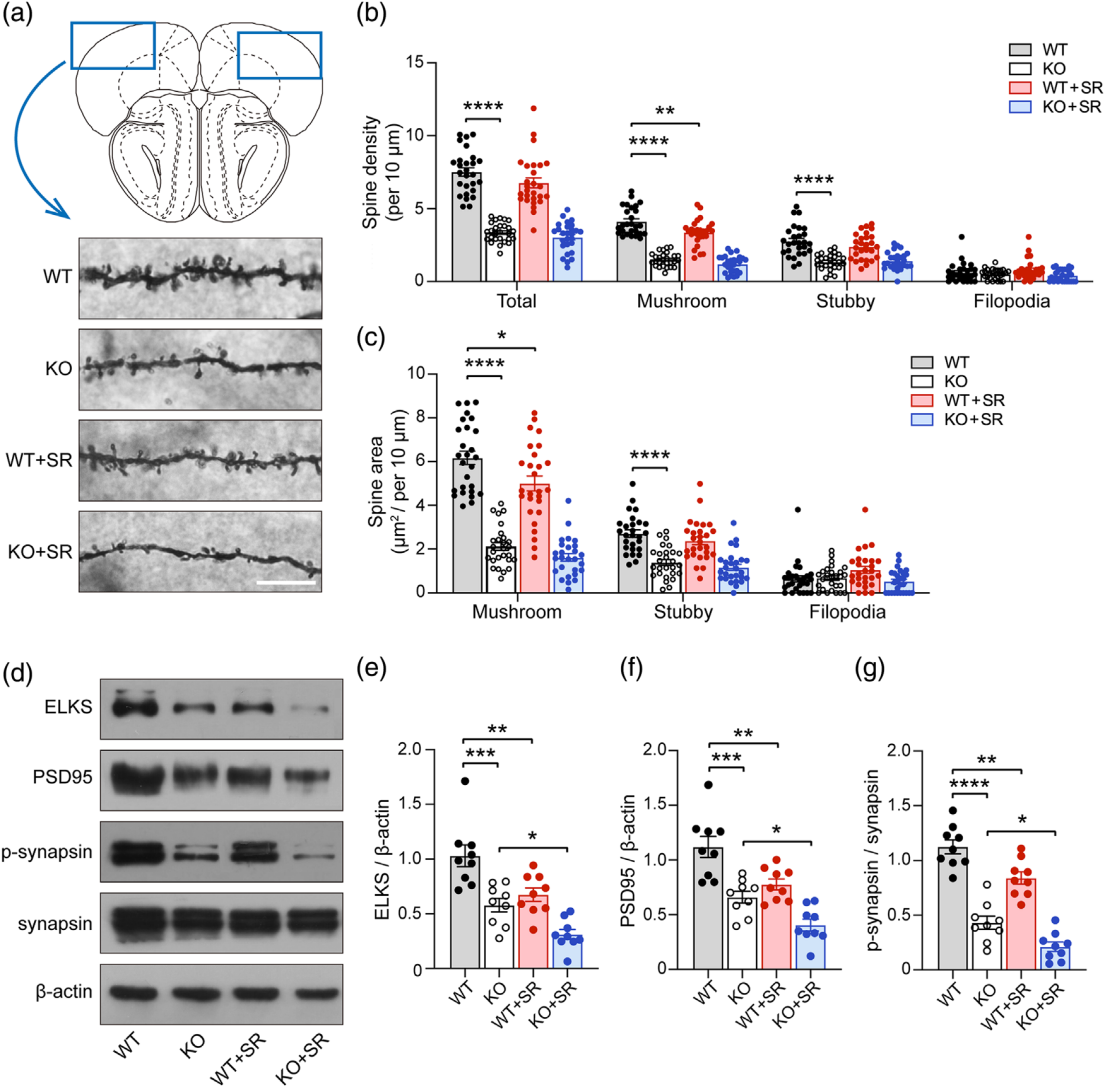
**The development of dendritic spines and synapses of four groups mice**: **(a)** the density and area of dendritic spines in prefrontal cortex (PFC) was assessed by Golgi stanning. Scale bar = 10 μm; **(b)** the density of total, mushroom, stubby, and filopodia; **(c)** the spine area of mushroom, stubby, and filopodia; **(d)** the schematic diagram of expression levels in ELKS, PSD95, and p‐synapsin, measured by Western blot; **(e–g)** the semiquantitative analysis of the expression of ELKS **(e)**, PSD95 **(f),** and p‐synapsin **(g)**. WT, wild type; KO, knockout; SR, sleep restriction. All groups: *n* = 3 (three times repeated). Data are the means ± SEM. *p < .05, **p < .01, ***p < .001, ****p < .0001.

The PFC is involved in higher order social, emotional, communication, cognitive function and development. Neural dysfunction in the PFC may contribute to cognitive impairments, lack of social interaction, and loss of inhibition of impulsivity (Courchesne et al., 2011). Abnormal PFC activity is frequently observed in patients with psychiatric disorders, such as schizophrenia (Thermenos et al., 2013), anxiety (Britton et al., 2013), autism (Amaral et al., 2008), and others (Drevets, 2000). It has been reported that δ‐catenin, encoded by *Ctnnd2*, is a component of the cadherin‐catenin cell adhesion complex that regulates spine and synapse morphogenesis during development (Arikkath et al., 2009; Matter et al., 2009). Accordingly, the structures of dendritic spines in the PFC of WT and KO mice, and WT and KO mice subjected to SR, were evaluated using Golgi staining. KO mice exhibited less total (*p* < .0001), mushroom (*p* < .0001), and stubby (*p* < .0001) type dendritic spine density than WT littermates (Figure 3a,b). In addition, KO mice exhibited decreased dendritic spine area of mushroom (*p* < .0001) and stubby (*p* < .0001) types compared to WT mice (Figure 3c). After SR, SR only decreased the dendritic spine density (*p =* .002) and area (*p* = .0102) of mushroom spine types in WT mice (Figure 3a–c). Dendritic spine density and mushroom area of KO mice were not further exacerbated by SR (Figure 3a–c).

The expressions of some synapse‐related proteins (ELKS, PSD95, and p‐synapsin) in the four groups of mice were assessed. As is shown in Figure 3d, the deletion of *Ctnnd2* reduced the expression of ELKS (*p* = .0004, Figure 3d,e), PSD95 (*p* = .0001, Figure 3d,f), and p‐synapsin (*p* < .0001, Figure 3d,g). Simultaneously, SR reduced the expression of ELKS (*p* = .0054, Figure 3d,e), PSD95 (*p* = .0047, Figure 3d,f), and p‐synapsin (*p* = .0055, Figure 3d,g) in WT mice. Moreover, compared with KO mice, SR further decreased the expression of ELKS (*p* = .0478, Figure 3d,e), PSD95 (*p* = .0487, Figure 3d,f), and p‐synapsin (*p* = .0462, Figure 3d,g) in KO mice. In summary, these results indicated that deletion of *Ctnnd2* in mice impaired the development of dendritic spines and synapses, and SR could exacerbate this damage in WT and *Ctnnd2* KO mice.

### 3.4 The PI3K/Akt‐mTOR signaling pathway is involved in the progress of SR‐induced impairment of phenotypes in WT and *Ctnnd2* KO mice

**FIGURE 4:**
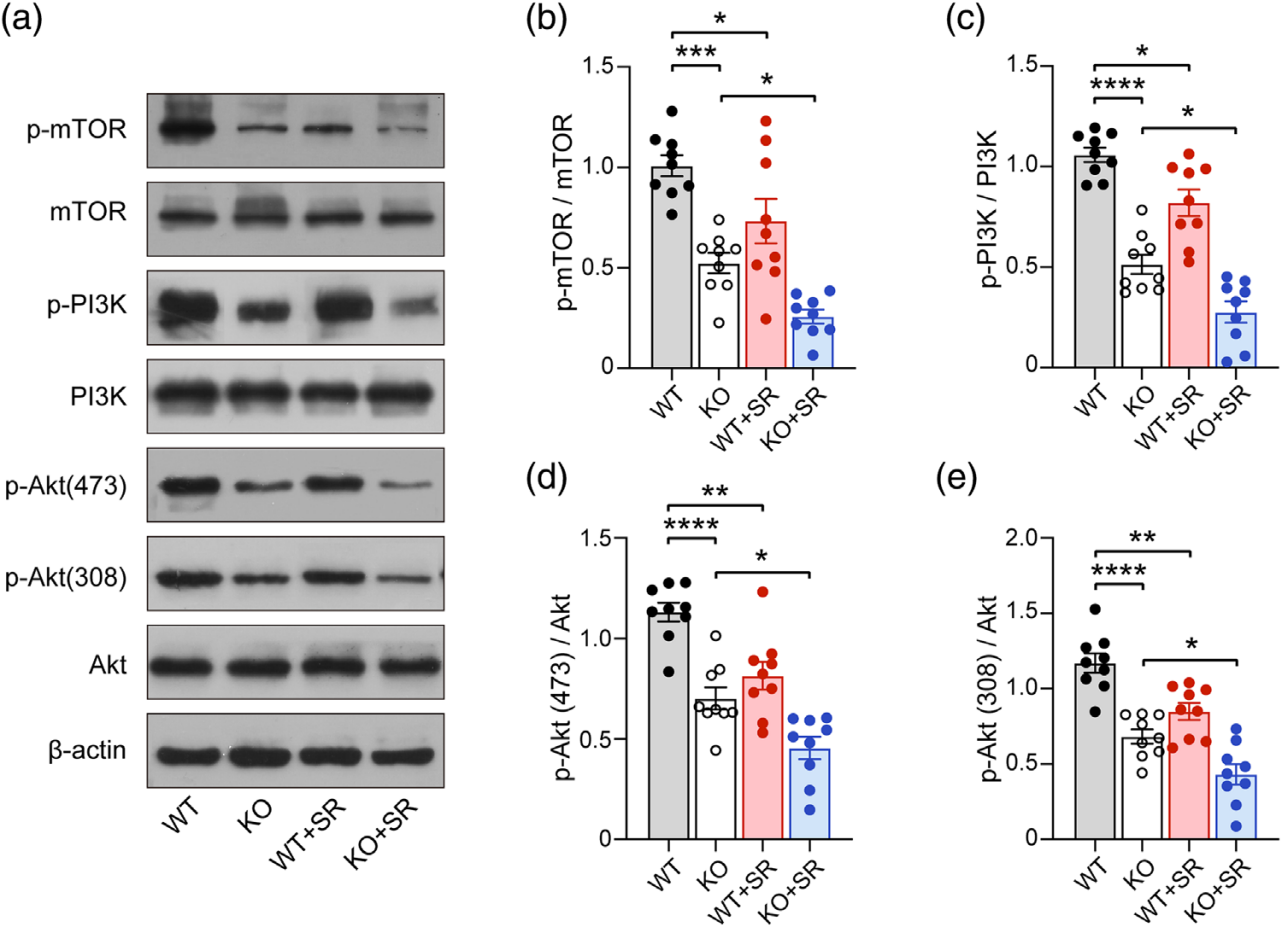
**The expression of PI3K/Akt‐mTOR signal pathway in four groups mice**: **(a)** the schematic diagram of expression levels in p‐mTOR, mTOR, p‐PIK3, PI3K, p‐Akt(473), p‐Aky(308), and Akt, measured by Western blot; **(b–e)** the semiquantitative analysis of the expression of p‐mTOR **(b)**, p‐PI3K **(c)**, p‐Akt(473) **(d),** and p‐Akt(308) **(e)**. WT, wild type; KO, knockout; SR, sleep restriction. All groups: *n* = 3 (three times repeated). Data are the means ± SEM. *p < .05, **p < .01, ***p < .001, ****p < .0001.

The PI3K/AKT‐mTOR signaling pathway is critical for synaptic plasticity and behavior in neurodevelopmental disorders arising from mutations, including cognitive dysfunction, autism, and intellectual disability (Borrie et al., 2017). Compared to their WT littermates, KO mice exhibited reduced expression of p‐mTOR (*p* = .0001, Figure 4a,b), p‐PI3K (*p* < .0001, Figure 4a,c), and p‐Akt (Ser 473: *p* < .0001, Thr 308: *p* < .0001, Figure 4a,d,e). In addition, an increasing number of researchers have recently focused on the molecular mechanism(s) of SR in mice, and some have indicated that SR could play roles in different situations by inhibiting the PI3K/Akt‐mTOR signaling pathway (Cao et al., 2019; Huang et al., 2018). The expressions of p‐mTOR (*p* = .0316, Figure 4a,b), p‐PI3K (*p* = .0135, Figure 4a,c), and p‐Akt (Ser 473: *p* = .0023, Thr 308: *p* = .003, Figure 4a,d,e) were consistently decreased by SR relative to that in WT mice. Compared to KO mice, SR further inhibited the expression of p‐mTOR (*p* = .0454, Figure 4a,b), p‐PI3K (*p* = .0139, Figure 4a,c), and p‐Akt (Ser 473: *p* = .0222, Thr 308: *p* = .0256, Figure 4a,d,e). These data suggest that the PI3K/Akt‐mTOR signaling pathway was not onlyinhibited by KO of *Ctnnd2* in mice but also involved in the progression of SR‐impairing phenotypes in WT and *Ctnnd2* KO mice.

## 4 DISCUSSION

The main finding of the present study was that deletion of the *Ctnnd2* gene in mice could lead to social interaction disorders, repetitive behaviors, diminished exploration behaviors, and defects in spatial cognition. In addition, the growth of dendritic spines and synapses in the PFC was significantly impaired in *Ctnnd2* KO mice. Furthermore, we found that some of these phenotypes in WT and *Ctnnd2* KO mice were also influenced by chronic SR, similarly to that observed in humans with autism. Finally, we observed that the PI3K/Akt‐mTOR signaling pathway was inhibited in *Ctnnd2* KO mice, and SR may regulate the development of dendritic spines and synapses mediated by the PI3K/Akt‐mTOR signaling pathway.

ASD is an abnormal neurodevelopmental condition characterized by early onset social communication difficulties and repetitive or stereotypical behaviors. For individuals with autism, comorbidities include anxiety, motor deficits (hypotonia, apraxia, or motor delay), intellectual disability, and sleep abnormalities (de la Torre‐Ubieta et al., 2016; Lai et al., 2019). Previous studies have reported that *CTNND2* is a candidate gene for intellectual disability, ASD, and growth retardation of dendritic spines and synapses (Arikkath et al., 2009; Baumert et al., 2020; Belcaro et al., 2015; Hofmeister et al., 2015; Turner et al., 2015; Yuan et al., 2015). In addition, overexpression of δ‐catenin, encoded by *Ctnnd2*, in mice could improve object recognition and social interaction, and reduce anxiety (Ryu et al., 2019). Accordingly, we had generated *Ctnnd2* KO mice using CRISPR‐Cas9 technology and demonstrated that the model animals exhibited autism‐like behaviors, and impaired learning and memory function (Wang et al., 2021). In this study, we demonstrated that *Ctnnd2* KO mice exhibited social interaction disorders assessed using a three‐chamber assay and direct social interaction test, more restrictive behaviors reflected by self‐grooming, reduced exploration behaviors according to open‐field test, and defects in spatial cognition according to MWM again.

Sleep disturbances are one of the most significant challenges experienced by patients with ASD, and often by extension the family members who provide care (Gisbert Gustemps et al., 2021). Regarding sleep disorders in the ASD population, studies have reported high prevalence rates, reaching 86% in childhood (Rzepecka et al., 2011). These difficulties are reported lifelong and, although the prevalence is not well established in adults, recent studies indicate a prevalence of 50%– 65% (Souders et al., 2017). It has been reported that such problems could further exacerbate ASD symptom severity and include more internalizing and externalizing behavioral problems, such as emotional reactivity and anxiety (Cohen et al., 2014; Hohn et al., 2019; Sikora et al., 2012). However, to date, research investigating the relationship between sleep profiles and behavioral problems in individuals with ASD is limited. Thus, in our study, after the investigation of that *Ctnnd2* KO mice exhibited ASD‐like behaviors, diminished exploration ability, and defects in cognition, to explore the effects of chronic SR on mice, we chose the method in which WT and *Ctnnd2* KO mice were subjected to 28 consecutive days of chronic SR as previously described (Lemons et al., 2018; Sare et al., 2016, 2019). In these studies, the response to stress was evaluated according to the sleep paradigm and serum corticosterone concentrations before and after SR, and the investigators observed that mice subjected to SR could be accustomed to long‐term chronic SR and exhibited normal stress similar to control mice (Bian et al., 2022; Sare et al., 2016, 2019). However, the chronic SR was administered at different developmental ages, and there was not a stress‐only control group in our study; thus, we had to consider that both SR and stress effects lead to exasperated autism‐like behaviors and cognition ability.

In the present study, chronic SR (PND21‐49) affected the social novel preference of WT and *Ctnnd2* KO mice in the three‐chamber assay; however, the results of this assay on SR in WT and *Ctnnd2* KO mice were not the same. In the novel preference test, after SR, WT mice spent less time interaction with Stranger 2 and KO mice spent more time interaction with Stranger 1. For WT mice, the finding of chronic SR in the social novel preference test was in general agreement with the recent studies of Bian et al. (2022) and Lord et al. (2022). The difference in response to SR in *Ctnnd2* KO mice may be largely not only due to the deficits in social novel preference but also related to the impairment of cognition rather than abnormal locomotion, as the total entries to distinct chambers in the three‐chamber assay were normal as in WT mice (Figure S1A,B). Although the stimulus mice were male conspecific and the mice were postpubertal and not gonadectomized, the results of the three‐chamber assay are reliable because they contain the target stranger within an inverted round wire cage that avoids potential confounders resulting from aggressive or sexual interactions (Moy et al., 2004). Simultaneously, in some published studies, mice 8–12 weeks of age were used in all behavioral studies (including the three‐chamber test) to avoid variability due to changes during adolescence (Gilbert et al., 2020). However, there was a limitation in the direct social interaction test, although there was no significant difference between the control mice and those subjected to SR. In addition, SR deteriorated the self‐restrictive behaviors of *Ctnnd2* KO mice in the open‐field test but had no influence on exploration ability and anxiety. However, increased time of self‐grooming in the open field may be a result of increased anxiety and, in the future studies, we need to further evaluate the repetitive behaviors using the marble burying test (Gilbert et al., 2020) and evaluate anxiety using the elevated plus‐maze test (Kraeuter et al., 2019). Therefore, our results demonstrated that SR influenced the social novel preference, but not sociability in mice, and deteriorated part of the autism‐like behaviors in *Ctnnd2* KO mice.

The literatures document that cognitive performance is also impaired by chronic SR and attention span, particularly prolonged attention, and affected by sleep deprivation in a dose‐dependent manner, especially in patients with ASD (Kansagra, 2020; Tsai et al., 2021). The results of the present study revealed that impairment in spatial memory ability following SR in WT mice was generally consistent with *Ctnnd2* KO mice. The swim speed from day 1 to 5 and the total exploration distance on day 6 showed no difference among the four groups mice, suggesting that all groups mice had no abnormal locomotor activity. Future research using chronic SR in *Ctnnd2* KO mice could explore the responses of different postnatal age periods’ SR and observe the condition after mouse sleep‐recovery.

Dendritic spines and synapses are largely responsible for receiving signals from other neurons and undergo numerous branching and elongation events throughout their development to establish an immense signal network and maintain normal function of the brain (Baumert et al., 2020). Altered synaptic structure and function are major hallmarks of ASD (Bagni & Zukin, 2019). δ‐catenin is required for proper dendrite development, as shRNA‐mediated knockdown of δ‐catenin leads to inhibition of both dendrite elongation and branching, whereas overexpression of δ‐catenin results in increased dendritic length and complexity (Arikkath et al., 2009). However, there are no published studies have reported the development of dendritic spines and synapses in *Ctnnd2* KO mice. In addition, we had a great interest in the observed dendritic spines density and synaptic protein levels in the PFC, one of the major brain regions associated with autism, in *Ctnnd2* KO mice. Fortunately, we observed that *Ctnnd2* KO mice exhibited reduced dendritic spines and decreased expression of synapse‐related proteins (ELKS, PSD95, and p‐synapsin), which may provide a molecular and synaptic structural basis for the behavioral characteristics of *Ctnnd2* KO mice.

Sleep is believed to consolidate the synaptic connections required for the encoding and retention of memory (Wang et al., 2011). Earlier findings have highlighted that short sleep deprivation results in more synaptic puncta and spines than normal sleep in the cortex or hippocampus, but the increased dendritic spines are immature and lack normal function (Areal et al., 2017; Wang et al., 2011). Chronic SR could not only reduce the total length of dendrites and density of spines across CA1 neurons (Noorafshan et al., 2018) but also decreased the expression of synaptic‐related proteins in the hippocampus (Kincheski et al., 2017). Similarly, in our study, after chronic SR, the density and area of dendritic spines (mushroom) in the PFC of WT mice decreased, and the density and area of dendritic spines in the PFC of *Ctnnd2* KO mice did not change. The expressions of synapse‐related proteins in the PFC of both WT and *Ctnnd2* KO mice were decreased. Therefore, the impaired cognitive ability observed in chronically sleep‐restricted WT and *Ctnnd2* KO mice could be explained by reduced dendritic spines and decreased expression of synaptic‐related proteins.

The PI3K/Akt‐mTOR pathway is a classical antiapoptotic and pro‐survival signaling pathway, and its activation is involved in the protective effects of various drugs on nerve cells. δ‐Catenin can interact with Shank3 and target postsynaptic sites (Hassani Nia et al., 2020). Shank3 is an upstream node of the PI3K/Akt signaling pathway and plays a pivotal role in mTOR signaling (Gropman, 2014). Therefore, the deletion of *Ctnnd2* may lead to dysfunction of the PI3K/Akt‐mTOR pathway. Recently, an increasing number of studies have focused on the relationship between the PI3K/Akt‐mTOR pathway and sleep. It has been reported that in the hippocampus of sleep‐deprived mice, microglia and astrocytes are activated, and the α7 nicotinic acetylcholine receptor and PI3K/Akt‐mTOR signaling pathways are inhibited, thus inducing downstream oxidative stress and inflammatory response. Administration of α7 nicotinic acetylcholine receptor agonists can reactivate the PI3K/Akt‐mTOR signaling pathway to reverse the pro‐inflammatory effects of sleep deprivation (Cao et al., 2019). In addition, the researchers have found that modafinil can improve the decreased learning and memory ability induced by sleep deprivation in mice by activating the PI3K/Akt‐mTOR signaling pathway in hippocampal neurons (Xue et al., 2019). In our study, deletion of *Ctnnd2* consistently inhibited the PI3K/Akt‐mTOR signaling pathway, and chronic SR further reduced the phosphorylation of PI3K, Akt, and mTOR. These results may imply that SR mediated deteriorated phenotypes mainly through the PI3K/Akt‐mTOR signaling pathway in *Ctnnd2* KO mice. Future studies aimed at gaining better understanding of the molecular correlates of our experimental mouse model may offer new treatment possibilities.

In this study, we investigated whether chronic SR could deteriorat part of the ASD‐like behaviors, cognition deficiency, and dysfunction of dendritic spines and synapses, but not influence anxiety and exploration behaviors. Moreover, the PI3K/Akt‐mTOR pathway is involved in the effects induced by chornic sleep‐deprived impaired phenotypes and may be the underlying molecular mechanism in *Ctnnd2* KO mice. In short, our study provides a further understanding of the role of disrupted sleep in *Ctnnd2* gene deletion induced autism on the evolution of neurodevelopmental disorders in mice, which may be consistently with patients with mutations in *CTNND2* gene.

## AUTHOR CONTRIBUTIONS

All listed authors participated meaningfully in this study and approved the submission of this manuscript. Man Xu, Xiaoya Wang, Shali Wang, Yingbo Li, Yihao Tao, and Shujiang Tan designed the experiment; Man Xu, Luyi Wang, Jing Deng, Yan Wang, and Yingbo Li collected and analyzed data, Man Xu and Sen Pan wrote the manuscript, and Luyi Wang and Ailing Liao prepared Figures 1, 2, 3, 4. All authors reviewed the manuscript.

## CONFLICT OF INTEREST STATEMENT

The authors declare that there are no conflict of interests.

### PEER REVIEW

The peer review history for this article is available at https://publons.com/publon/10.1002/brb3.3075

## Supporting information


**Figure S1 The results of locomotor of four group mice in three‐chamber assay**. (A and B) Schematic presentation and statistical analysis of total number of crossings in the first 10 min (A) and second 10 min (B).
