# Supplementary figures and images for: Effects of chronic sleep restriction on the neuro‐phenotypes of Ctnnd2 knockout mice
Source: Brain Behav. 2023 May 24;13(7):e3075. doi: 10.1002/brb3.3075 (PMC10338785; doi:10.1002/brb3.3075)

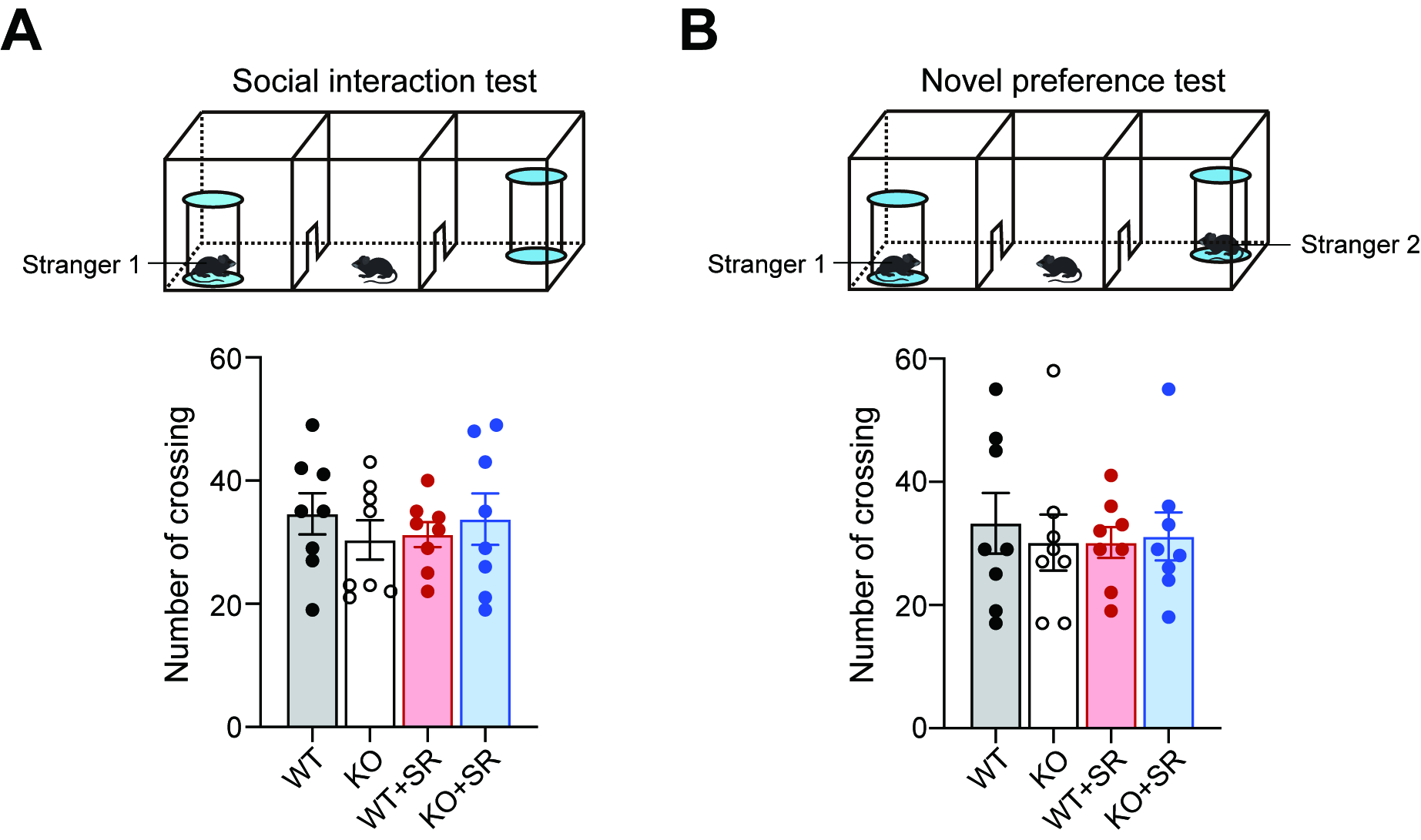

Supplement: Supplementary file 1 — Figure S1 The results of locomotor of four group mice in three‐chamber assay. (A and B) Schematic presentation and statistical analysis of total number of crossings in the first 10 min (A) and second 10 min (B). [file BRB3-13-e3075-s001.tif]
